# Stereological study of the effect of ginger's alcoholic extract on the testis in busulfan-induced infertility in rats

**Published:** 2013-06

**Authors:** Hossein Bordbar, Tahereh Esmaeilpour, Farzaneh Dehghani, Mohammad Reza Panjehshahin

**Affiliations:** 1 *Department of Anatomy, Shiraz University of Medical Sciences, Shiraz, Iran.*; 2 *Histomorphometry, Stereology Research Center, Shiraz University of Medical Sciences, Shiraz, Iran.*; 3 *Department of Pharmacology, Shiraz University of Medical Sciences, Shiraz, Iran.*

**Keywords:** *Busulfan*, *Cavalieri method*, *Ginger extract*

## Abstract

**Background: **In traditional medicine zingiber officinale used to regulate female menstural cycle and treat male infertility. Recent studies have suggested the possible role of ginger extract in improving the testicular damage of busulfan.

**Objective:** The aim of this study was to evaluate the effects of zingiber officinale on the sperm parameters, testosterone level and the volume of the testes and seminiferous tubules by stereological methods.

**Materials and Methods: **Fifty rats were divided into four groups. All the rats were given a single intraperitoneally injection of 5mg/kg busulfan solution. The first group was kept as busulfan control, while the other groups were orally administrated ginger extract in graded doses of 50, 100 and 150mg/kg b.wt, for 48 consecutive days. At the end, all animals were anesthetized and their testes and vas deference were removed, fixed, embedded, and stained. The volume of testes and seminiferous tubules were estimated by cavalieri methods.

**Results:** The result showed, that zingiber officinale increased the volumes of seminiferous tubule in 100mg/kg treated group compared to control group. Sperm count (706×10^5^ and 682×10^5^) and the level of testosterone (50.90 ng/mL and 54.10 ng/mL) enhanced in 100 mg/kg and 150 mg/kg treated groups compared to control group (p=0.00).

**Conclusion:** It seems that zingiber officinale stimulate male reproductive system in induce busulfan infertility.

## Introduction

Many factors such as life style, drugs, toxicants, nutritional imbalances, and infections lead to infertility (1). Some drugs such as cisplatin and busulfan induce reproductive toxicity. Busulfan is used to treat chronic myelogenous leukemia and blood disorders (2, 3). It has been revealed that busulfan decreases sperm motility, viability and count. This drug reduces testicular weight, and diameter (4, 5). Using medicinal plants in order to treat infertility has its roots in ancient cultures. It has been reported that some plants stimulate or inhibit testicular functions (6). 

Various natural products of plant, such as Korean ginseng, Cinnamon, Roselle, Phoenix dactylifera pollen, Ginger, Actinidia chinensis, and Foeniculum valgare affected different aspects of male fertility (7, 8). One research indicated the toxic effect of ginger extract on human sperm parameters in vitro (9, 10). However, other studies showed that ginger increases sperm count as well as motility, and has a stimulatory effect on serum testosterone level (11, 12). Traditionally, ginger rhizome was used in Iran for enhancing male sexuality, regulating female menstrual cycle, and also reducing painful menstrual periods (13, 14). 

A great number of studies have reported that watery or alcoholic ginger extract can increase the weight of testis and accessory reproductive glands (15, 16). There is a direct relationship between weight and volume; therefore, applications of stereological studies are necessary in order to precisely measurment the induced-atrophy by busulfan or the recovery by ginger. Nowadays, stereological studies are highly important and can be applied in quantitative studies of different parameters of testis.

The present study aims to investigate the effects of different doses of ginger root extract in male induced sterility rats through a stereological study.

## Materials and methods

**Plant material and extraction procedure**

Zingiber officinale L. (Ginger, Family Zingiberaceae) dried roots was obtained from a local company (Gol Darou, Isfahan, Iran). Authentication of the plant was carried out by a botany specialist and was identified by Voucher Number: 86.1133.1. The methanolic extract was prepared using simple percolation method. Briefly, 200 g of dry Z. ofﬁcinale roots were soaked in 1400 ml of 70% methyl alcohol for 3 days. The infusions were filtered through the Wattman filter no. 40 and methanol was evaporated using a rotator evaporator system. The remaining extract was dried in a desiccator (Labteron, Tehran, Iran). After 24 hours, 25 grams of 12.5% crystallized extract were prepared. 

**Animals**

Adult male Sprague Dawley rats (n=50) weighing between 200-250 g were housed in a well-ventilated animal house under standard conditions, the relative temperature of 22±2^o^C, 12h/12h light/dark cycle, and the relative humidity of 60-80%. They were fed with standard diet and water. Only healthy animals were selected for the experiment. This study was designed by the Ethics Committee, Shiraz University of Medical Sciences, Shiraz, Iran. This investigation was designed as experimental study.

**Experimental design**

The animals were randomly categorized into four equal groups. The rats in all the groups were given a single intraperitoneally injection of 5 mg/kg busulfan solution (United pharm, Korea). The first group was kept as the Busulfan control, while the other groups orally received the ginger extract. One day after the Busulfan injection, the rats were orally given the ginger extract in graded doses of 50, 100 and 150 mg/kg b.wt for 48 consecutive days (5, 17). At the end of the experiment, blood samples were taken from their tail (1mL) in order to determine the testosterone level both before and 48 days after the treatment. 

Then, the rats were weighted and then scarified using anesthetic diethyl ether. Testes and vas deference of each animal were removed. Semen sample were collected from vas deference for sperm analysis. Then, the testes were weighted (ScalTEC SPO 51- Germany), fixed in Bouin's fixative, dehydrated, and embedded in paraffin. Afterwards, the testis parameters (length, width) were measured by a digital sliding caliper (Mitutoyo, CD-12"CP, apan). 

**Blood sampling and hormone assay**

Blood samples which were collected from the rats’ tail vein both before and after the treatment were centrifuged (Eppendorf, 5804-R, Germany) at 4^o^C for 10 minutes at 250 g. Also, the obtained serum was stored at -20^o^C until the biochemical analysis. Serum testosterone concentration was determined using the radioimmunoassay method (RIA) by a kit (Immunotech, Czech Republic). This method is based on the competitive binding principle. Radioactive iodine125 (I ^125^) labeled testosterone was used as the labeled antigen. The bound radioactivity was determined using the gamma counter. Inter assay coefficient of variation for testosterone was 15%, while Intra assay coefficient of variation for testosterone was 14.8%. The analytic sensitivity for testosterone was 0.025 ng/ml.

**Sperm analysis**

After treatment, the spermatozoa were obtained from final 1 cm of vas deference which had been put in the HBSS (Hanks Balanced Salt Solution) and was microscopically evaluated for sperm count and motility. At least 100 rapid progressive motility sperm were analyzed in 10 fields by invert microscopy. Sperm cell counts were determined using the standard hemocytometer method.

**Stereological study**

Volume density and volumes of the testis, seminiferous tubules and interstitial tissue were assessed by the Cavalieri method. The Cavalieri method (Howard and Reed, 2005) was used as an estimator of the testis volume. Thus, eight to eleven sections were selected using a systematic sampling design and a random start for stereological estimations. Each sampled section was analyzed using a video-microscopy system made up of a microscope (Nikon, E-200, Japan) which was linked to a video camera (SONY, SSC Dc 18P, Japan), a P4 PC computer, and a LG monitor (795 FT plus) in order to determine the parameters. By means of stereology software designed at our lab, the stereological probe (points) was superimposed upon the images of the tissue sections and was viewed on the monitor. The testis volume was estimated using the following formula:

$V\left(\mathrm{total}\right)=\sum \mathrm{p.}(a/\mathrm{p0.d}$

Where the “V (total)” was the gland volume, “ΣP” was the sum of the points falling on the section profile, “a/p” was the area associated with each point at the level of tissue, and “d” was the distance between the sampled sections (18). 

**Statistical analysis**

Data were expressed as mean±SD. Also, statistical analysis were carried out through one way ANOVA, t-test and LSD tests. P-values <0.05 were considered as statistically significant. The statistical analysis were done by the SPSS statistical software (version 15).

## Results

**Volume of testis and seminiferous tubules**

Stereological examination of the total volume (mm^3^) of the testis and the volume (mm^3^) of the seminiferous as well as the interstitial tissue are shown in Table I. Oral administration of ginger extract at three given doses significantly increased the total volume of the testis compared to the busulfan control group (p=0.00). However, at dose of 150 mg/kg b.wt there was a significant decrease in comparison to both ginger administrated groups. 

The data showed that administration of 50 and 100 mg/kg b.wt ginger extract significantly increased the seminiferous tubules volume compared to the busulfan control group (p=0.00). Administration of the dose of 150 mg/kg b.wt of the ginger extract didn't cause any significant difference compared to busulfan control group (p=0.06).

**Volume of interstitial tissue**

Significant changes in the interstitial tissue volume of the testis were found in rats treated with all doses of ginger extract in comparison to the busulfan control group (p=0.00). Moreover, the administration of 50 mg/kg b.wt of the extract had led to the maximum effect.

**Testosterone level**

The testosterone level (ng/ml), sperm count and motility (%) are presented in Table II. Radioimmunoassay of serum samples as shown in table II, revealed that administration of either100 or 150 mg/kg b.wt ginger extract significantly increased the testosterone level in comparison to the busulfan control group, while 50 mg/kg b.wt concentration of the extract didn't have the same effect (p=0.15). When comparison applied within groups, the testosterone level was significantly increased with higher concentration of the ginger extract.

**Sperm count and motility**

After different doses of ginger extract were administrated, sperm count and motility significantly increased compared to the busulfan control group (p=0.00). This intensification of sperm motility in ginger-treated groups is dose-dependent with the maximum effect occurring at the high dose (150 mg/kg b.wt).

**Table I:** The total volume of the testis, testicular seminiferous and interstitial tissue volume of male rats exposed to ginger extract (n=50)

	**Total volume (mm** ^3^ **)**	**Seminiferous tubule volume (mm** ^3^ **)**	**Interstitial tissue (mm** ^3^ **)**	**p-value volume (t-test)**
Busulfan Control	418.17 ± 24.58	83.97 ± 2.42	64.30 ± 12.4	0.00
Busulfan+50 mg/kg GE	1015.48± 344.99 ^ a^	90.89 ± 4.74 ^ a^	111.50 ± 35.98^ a^	0.00
Busulfan+100 mg/kg GE	1183.85 ± 213.37 ^a^	91.43 ± 2.42^ a^	98.28 ± 23.42^ a^	0.00
Busulfan+150 mg/kg GE	755.63 ± 149.77^a, b ,c^	87.32 ± 2.81^ c^	96.63 ± 34.40 ^ a^	0.06

Statistical analyses were carried out through one way ANOVA, t-test and LSD tests.

P-values<0.05 were considered as statistically significant.

The number of animals per group was ten rats. Data are presented as means±SD (95% CI for mean). GE: Ginger Extract.

**Table II:** The testosterone level, sperm count and motility of male rats exposed to ginger extract (n=50)

	**Testosterone Level (ng/mL)**		**Sperm count**	**Progressive sperm motility (%)**	**P-value** **(t-test)**
	**Before treatment**	**After treatment**	**After treatment**	**After treatment**	
Busulfan Control	2.00±1.53	0.92 ± 0.50	29.6×10^5^ ± 21809233.52	36.10 ± 17.91	0.00
Busulfan + 50 mg/kg GE	1.99±1.50	1.82 ± 1.71	698×10^5^±20853137.25^ a^	49.50 ± 7.62 ^a^	0.00
Busulfan + 100 mg/kg GE	1.08±0.54	2.76 ± 1.90^ a, e^	706×10^5^± 13442883.29^ a^	50.90 ± 8.14^ a^	0.15
Busulfan + 150 mg/kg GE	1.22±0.90	2.56 ± 1.14 ^a, e^	682×10^5^± 15042458.43 ^a^	54.10 ± 9.69 ^a^	0.15

Statistical analyses were carried out through one way ANOVA, t-test and LSD tests.

P-values<0.05 were considered as statistically significant.

For legend, refer to Table I.

GE: Ginger Extract.

P <0.05 considered as statistical significant.

## Discussion

The present study aimed to determine whether ginger extract improves partial infertility in male rats induced by busulfan using quantitative measurements. Many studies have described the decreasing effect of busulfan on sperm count and motility as well as its reducing effect on testicular weight and diameter (3, 5). It seems that the increasing production of free radicals and oxidative stress by busulfan results in DNA destruction and induces apoptosis and infertility (19-20). To avoid of toxic and side effect of busulfan on male reproductive system, several drugs and material should be consumed. It has been shown that combined treatment of busulfan, melatonin and liver growth factor reduces the busulfan’s side effects as well as its endocrine disorders (21, 22). Many investigations showed that some plant extract such as ginger have protective and increasing effects on spermatogenic cells and diameter of seminiferous tubules (23).

In this study, ginger is revealed to increase the testicular and the seminiferous volume, and sperm count as well as motility at 100 mg/kg ginger extract. Stereological examinations showed that treating animals with ginger extract decreased the testicular damages induced by busulfan. The data of the volume of the seminiferous tubules showed that exposing the rats to ginger extract significantly increased the volume. However, it was not significant in comparison to the control group. In all the three experimental groups, the total testicular and the interstitial tissue volumes significantly increased compared to the control group.

It seems that the increased total volume of the testis is due to the increasing volume of both seminiferous tubules and interstitial tissue containing Leydig cells. In line with our results, it was reported that ginger could overcome the reproductive toxicity induced by metriam and stimulate the seminiferous to be compact to each other with few or no apoptosis. 

It was also demonstrated the significant increase in the diameter of the tubules and their spermatogenic cells (24). In another study, the protective influence of ginger against toxicity produced by aluminum chloride has been shown. Histopathological examination showed the improvement of degenerative changes of the seminiferous tubules after ginger consumption for 60 consecutive days (25). The reducing effect could be seen in administration of the high dose of ginger in this study. Overall, it may be concluded that all the above mentioned testicular parameters are dose-dependent and increasing the ginger concentration may exert a reducing effect and produce a reproductive toxicity. In agreement with the results of the present study, Jorsaraei *et al* reported that changes in motility and morphological profiles of the sperm after usage of ginger in vitro are dose-dependent (9).

In the present study, the beneficial effect of ginger extract on sperm parameter has been well documented. All administered doses of ginger extract are related to the improvement of sperm motility and quantity in comparison to the control group as shown in the table II. In addition, the results also revealed that administration of higher doses of ginger extract had a significant effect on the serum testosterone level when compared to the control group as well as within groups. Our findings are in line with those who confirmed that the testosterone level increased significantly at dose of 100 mg/kg b.wt of ginger powder. 

They also demonstrated the improvement in sperm percentage, viability and motility when ginger was consumed at both 50 and 100 mg/kg b.wt concentrations (10). They also reported the increase in the level of the testosterone and the testes’ weight due to the androgenic activity of ginger (26). The protective role of ginger against reproductive destructive materials including cisplatin gentamicin and arsenite is also confirmed in a great number of investigations (2, 27). 

Increase of the interstitial tissue volume and the testosterone level might have resulted from the Leydig cells which presented in the interstitial tissue and secret testosterone. It may be explained that enhancement of volume or number of Leydig cells results elevation of the testosterone level. It has been established that ginger has antioxidant properties which can be contributed to its protective components such as zingerone, gingerdiole, zingirbrene, gingerls and shagaols. These compounds prevent DNA damages and destruction of genome induced by H_2_O_2_ (14, 20). It would be expected that ginger cause an increase in the spermatogenesis number and seminiferous tubules volume by preventing apoptosis in the spermatogenesis process. 

## Conclusion

In conclusion, results of this study revealed that ginger extract increased testicular volume and reduced the side effects of busulfan, but it is dose-dependent. Therefore, according to the results, ginger administration may be useful for patients who have undergone chemotherapy with induced sterility. To access better results more investigations should be established.
